# APPROACHES TO RECRUITING NON-ENGLISH-SPEAKING LATE-LIFE CHINESE IMMIGRANTS FOR HEALTHY AGING STUDIES

**DOI:** 10.1093/geroni/igz038.1330

**Published:** 2019-11-08

**Authors:** Wenjun Li, Linda Churchill, Jie Cheng, Rachel Siden, Annabella Aguirre

**Affiliations:** University of Massachusetts Medical School, Worcester, Massachusetts, United States

## Abstract

Non-English Speaking late-life Chinese immigrants are hard to reach. We developed a staged, multi-facet, community-engaged approach to recruiting participants for aging research. We first used a direct mail campaign targeting neighborhoods with high concentrations of racial minorities, and sent mails to households with a possible Chinese family name. Invitational letter, interest survey and flyers are printed in traditional and simplified Chinese using large font. Flyers include a colorful graphic portraying diverse racial background. Prior to the mailing, we presented the study at senior centers, faith-based organizations, community centers and bingos that hosted higher rates of minority older adults. We posted study materials in Chinese language schools and Chinese “WeChat” groups. We also encouraged current participants to “tell a friend”. Chinese-English bilingual staff are trained and certified as recruiters. Within two months, over 50 participants from diverse neighborhoods are recruited. Our community-engaged, linguistically and culturally appropriate approach has been highly effective.

